# L-Threonine-Derived Biodegradable Polyurethane Nanoparticles for Sustained Carboplatin Release

**DOI:** 10.3390/pharmaceutics17010028

**Published:** 2024-12-27

**Authors:** Seoeun Oh, Soo-Yong Park, Hyung Il Seo, Ildoo Chung

**Affiliations:** 1 Department of Polymer Science and Engineering, Pusan National University, Busan 46241, Republic of Korea; 2 Department of Surgery, Biomedical Research Institute, Pusan National University Hospital, Pusan National University School of Medicine, Busan 49241, Republic of Korea; seohi71@pusan.ac.kr

**Keywords:** biodegradable, double emulsion, nanoparticles, carboplatin

## Abstract

**Background and objectives:** The use of polymeric nanoparticles (NPs) in drug delivery systems offers the advantages of enhancing drug efficacy and minimizing side effects; **Methods:** In this study, L-threonine polyurethane (LTPU) NPs have been fabricated by water-in-oil-in-water emulsion and solvent evaporation using biodegradable and biocompatible LTPU. This polymer was pre-synthesized through the use of an amino acid-based chain extender, desaminotyrosyl L-threonine hexyl ester (DLTHE), where urethane bonds are formed by poly(lactic acid)-poly(ethylene glycol)-poly(lactic acid) (PLA-PEG-PLA) triblock copolymer and 1,6-hexamethylene diisocyanate (HDI). LTPU is designed to be degraded by hydrolysis and enzymatic activity due to the presence of ester bonds and peptide bonds within the polymer backbone. LTPU NPs were fabricated by water-in-oil-in-water double emulsion solvent evaporation methods; **Results:** The polymerization of LTPU was confirmed by ^1^H-NMR, ^13^C-NMR, and FT-IR spectroscopies. The molecular weights and polydispersity, determined with GPC, were 28,800 g/mol and 1.46, respectively. The morphology and size of NPs, characterized by DLS, FE-SEM, TEM, and confocal microscopy, showed smooth and spherical particles with diameters less than 200 nm; **Conclusions:** In addition, the drug loading, encapsulation efficiency, and drug release profiles, using UV-Vis spectroscopy, showed the highest encapsulation efficiency with 2.5% carboplatin and sustained release profile.

## 1. Introduction

The application of a drug delivery system plays a critical role in the efficacy and safety of drug therapy. With the advancement of medical technology, researchers are designing novel drug delivery systems that can improve drug bioavailability and selectivity while reducing side effects [1,2,3]. This progress is essential because many drugs have limitations in their route of delivery, biodistribution, and pharmacokinetics, which can compromise the drug’s therapeutic potential [4,5]. Therefore, research on delivery systems is necessary to advance drug development and clinical practice.

Polymers are commonly used to fabricate drug delivery systems because of their numerous benefits, including the ability to control the release of drugs through degradation [6,7,8,9], which can lead to more consistent drug levels in the body, resulting in improved drug efficacy. In addition, drug delivery systems using polymers can protect drugs from degradation and elimination, further improving their efficacy [7]. Another important benefit of polymer-based drug delivery systems is the ability to reduce side effects [6,10,11]. Polymers can be modified to conjugate molecules that target specific cells or tissues, reducing the exposure of healthy tissues to the drug [12,13].

The degradation of polymers can be controlled by the introduction of hydrolyzable bonds in the polymer backbone; varying the types and number of these bonds can regulate the degradation. Many biodegradable polymers are synthesized using amide linkages such as polyamide, polyurethane, polyurea, or ester linkages, including PLGA, PGA, PCL, polyanhydride, etc. [14,15,16,17,18,19,20,21,22,23]. Faster degradation is typically achieved in copolymers containing both ester and amide bonds when a higher proportion of ester bonds is present [24,25,26,27,28,29,30,31,32,33]. Although the importance of PLGA and other ester-based polymers cannot be overstated, the biological disadvantages of PLGA have been well documented, such as the lowering of the local pH and the activation of the foreign body responsive. Therefore, our goal is to develop an alternative biodegradable polymer with an enzymatically degrading mechanism in which some of the degradation products can be metabolized by the local cells. We previously reported a study investigating the therapeutic effects of microRNA-378a on liver fibrosis, utilizing L-tyrosine polyurethane (LTU) nanoparticles as gene carriers to deliver microRNA-378a to an animal model. Furthermore, the results directly supported our hypotheses regarding the mechanism of liver fibrosis and highlighted a promising approach for using microRNA-378a as both a biomarker of liver pathogenesis and a therapeutic agent for liver disease, while also providing indirect evidence of the non-toxic and non-carcinogenic properties of amino acid-based polyurethanes [18,19]. L-threonine was chosen as the building block of a novel biodegradable polymer because of its role in protein synthesis, serving as a precursor for various catabolic pathways, contributing to improved muscle control, and functioning as a signaling molecule. L-threonine also has a dipole moment, although its overall charge is zero, which could be advantageous for enhanced loading for drugs with charge groups when preparing drug delivery systems.

The application of delivery systems for cancer therapies offers numerous benefits, with increased drug efficacy being chief among them, leading to reduced drug dosages and fewer side effects [34,35,36,37]. Cancer tissues in the body typically have relatively loose vascular epithelial cell gaps and reduced lymphoid tissue compared to normal blood vessels, allowing drugs or drug carriers, such as nanoparticles (NPs), to penetrate into the cancer tissues. This phenomenon is known as the enhanced permeation and retention (EPR) effect, and passive targeting NPs can accumulate within tumors [38,39,40].

In this study, a novel biodegradable polyurethane, which can undergo degradation through both hydrolysis and enzymatic mechanisms, was synthesized by initially conjugating desaminotyrosine with L-threonine via a peptide bond (DLTHE). In the second step, DLTHE was reacted with a polyester diol and an isocyanate, forming urethane linkages (LTPU). Since PLGA nanoparticles loaded with carboplatin have been fabricated to investigate biological and antitumor activities, this study also explores the feasibility of applying LTPU as a drug delivery platform for the sustained release of carboplatin [41]. Carboplatin-loaded LTPU NPs were formulated and characterized for their size, morphology, encapsulation efficiency, in vitro drug release, and physiological stability.

## 2. Materials and Methods

### 2.1. Materials

L-threonine, 1-hexanol, sodium chloride (NaCl), desaminotyrosine (DAT), anhydrous toluene, tin(II) 2-ethyl hexanoate (Sn(Oct)_2_), anhydrous N, N-dimethylformamide (DMF), poly(vinyl alcohol) (PVA, Mw 30,000–70,000), fluorescein isothiocyanate (FITC) and phosphate-buffered saline (PBS), dimethyl sulfoxide-d_6_ (DMSO-d_6_), and chloroform-d (CDCl_3_) were purchased from Sigma Aldrich (Milwaukee, WI, USA). Thionyl chloride, sodium carbonate (Na_2_CO_3_), acetone, tetrahydrofuran (THF), hydrochloric acid (HCl, 36.5%), 1-ethyl-3-(3-dimethylaminopropyl)-carbodiimide hydrochloride (EDC-HCl), di-ethyl ether, dichloromethane (DCM), hexamethylene diisocyanate (HDI), chloroform, and ethyl acetate were purchased from Daejung Chemical and Metal (Siheung, Republic of Korea). Poly(ethylene glycol) (PEG, Mw ~400), L-(−)-lactide, dibutyltin dilaurate (DBTDL), and carboplatin were purchased from Tokyo Chemical Industry (TCI) (Tokyo, Japan), and magnesium sulfate (MgSO_4_) was purchased from Yakuri Pure Chemicals (Kyoto, Japan). All other chemicals were of reagent grade and used without further purification.

### 2.2. Synthesis of LTPU

#### 2.2.1. L-Threonine Hexyl Ester (LTHE)

**Figure 1:**
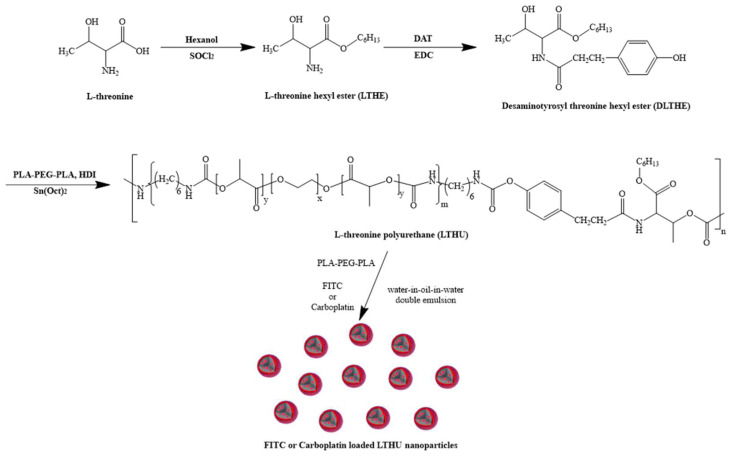
Fabrication of biodegradable FITC-loaded NPs and carboplatin-loaded LTPU NPs (CLNPs).

L-threonine (0.075 mol) and n-hexanol (150 mL, 1.1925 mol) were taken into a 250 mL three-neck round bottom flask. The reaction mixture was stirred at 450 rpm, 0 °C, under a nitrogen atmosphere. Thionyl chloride (8.1 mL, 0.1125 mol) was added dropwise to the reactor for 20 min through a dropping funnel, and the stirring speed was increased to 1150 rpm. The reaction mixture was allowed to react for an additional 15 h at 80 °C with continuous stirring at 400 rpm. After the reaction was completed, the reaction mixture was cooled down to room temperature for 10 min with continuous stirring, and then the unreacted reactants were removed by a rotary evaporator. A 0.5 M Na_2_CO_3_ aqueous solution of 5 mL was added to the remaining solution to neutralize pH, and then the solution was filtered using acetone as a solvent to remove salts. The solvent was removed by a rotary evaporator and dried in a vacuum oven for 2 days (Figure 1).

#### 2.2.2. Desaminotyrosyl L-Threonine Hexyl Ester (DLTHE)

Desaminotyrosine (0.06 mol), EDC-HCl (0.045 mol), and dried THF (60 mL) were taken into a 250 mL three-neck round bottom flask at 0 °C under a nitrogen atmosphere and stirred at 400 rpm. After 5 min, LTHE (12.18 g, 0.06 mol) in dry THF 20 mL solution was added to the reactor. After the reaction mixture was stirred for 2 h at 0 °C, the reaction was further continued at 34 °C with continuous stirring at 220 rpm for 14 h. After the reaction was completed, the reaction mixture was poured into cold DI water (150 mL). The aqueous solution was extracted three times using DCM (60 mL) to remove urea as a byproduct. The collected organic solution was then extracted three times using a 0.1 M HCl solution (60 mL) to remove unreacted LTHE. It was subsequently washed with a 0.1 M Na_2_CO_3_ aqueous solution (60 mL) and 21 g/100 mL NaCl solution (60 mL) to neutralize the pH and remove unreacted DAT, respectively. Anhydrous MgSO_4_ was added to the solution and stirred for 1 h at 400 rpm to remove traces of moisture. After 1 h, the solution was filtered, and the solvent was removed by a rotary evaporator, and then it was dried under a vacuum oven for 2 days.

#### 2.2.3. PLA-PEG-PLA Triblock Copolymer

The triblock copolymer was synthesized and characterized following our previous method with minor modifications [18]. Briefly, PEG 400 (6 mmol) was added to a three-neck round-bottom flask and dissolved in anhydrous toluene (200 mL). The solution was then refluxed at 140 °C for 2 h under a nitrogen atmosphere with continuous stirring at 400 rpm. 250 mg of tin(II) 2-ethylhexanoate (Sn(Oct)_2_) in anhydrous toluene (2 mL) was added to the above reaction solution. After 10 min, L-(−)-lactide (14.688 g, 102 mmol) was added, and the reaction was allowed to continue for 20 h under a nitrogen atmosphere. After the reaction was stirred for an additional 20 min at room temperature, the solvent was evaporated, and the remaining solid was dissolved in 15 mL of DCM, followed by precipitation in cold diethyl ether. The precipitate was collected by filtration and then dried in a vacuum oven for 2 days.

#### 2.2.4. LTPU Polymerization

PLA-PEG-PLA (2 mmol), HDI (4 mmol), and dibutyltin dilaurate (DBTDL, 70 μL) were added to a three-neck round bottom flask and dissolved in anhydrous DMF (20 mL). The mixture was then reacted for 3 h at 70 °C under a nitrogen atmosphere with continuous stirring at 400 rpm. After DLTHE (2 mmol) was dissolved in anhydrous DMF (2 mL) and added to the above reaction mixture, the reaction was continued for an additional 4 h under a nitrogen atmosphere, followed by 10 min at room temperature. The LTPU was then collected by precipitation, washed several times with cold DI water, and freeze-dried for 3 days.

### 2.3. Fabrication of Blank and FITC-Loaded LTPU NPs

As shown in Table 1, blank NPs and FITC-loaded LTPU NPs were prepared by the water-in-oil-in-water (W/O/W) double emulsion solvent evaporation method using FITC solution or distilled water. FITC was encapsulated to confirm the structure of nanoparticles through the loading of the drug in water-in-oil-in-water double emulsion processes. LTPU and PLA-PEG-PLA in ethyl acetate, along with DI water with or without FITC, for either FITC LTPU nanoparticles or blank nanoparticles, were added to a 50 mL conical tube. An emulsion was formed by stirring at 19,700 rpm using a homogenizer for 1 min and then poured into a beaker. After reducing the stirring rate to 12,700 rpm, 50 mL of a 5% (*v*/*v*) PVA solution was added to the beaker, and the mixture was stirred for an additional 15 h at the lower stirring rate. The emulsion was centrifuged for 20 min at 4 °C at 14,000 rpm to collect the nanoparticles (NPs). The NPs were subsequently washed twice with deionized (DI) water, followed by centrifugation for an additional 20 min at 4 °C and 14,000 rpm. Finally, they were freeze-dried for 3 days.

### 2.4. Fabrication of Carboplatin-Loaded LTPU NPs

All fabrication procedures for carboplatin-loaded LTPU NPs (CLNPs) are the same as those for FITC-loaded LTPU NPs, water-in-oil-in-water (W/O/W) double emulsion solvent evaporation method, except for the substitution of carboplatin solution in place of FITC solution. Carboplatin concentrations of 2.5%, 5%, 7.5%, and 10% in DI water were prepared and then encapsulated into LTPU NPs to assess the optimized carboplatin-loading percentage.

Each weight of carboplatin was dissolved in 1 mL of distilled water. The particles were evaluated to verify the optimized carboplatin quantity used relative to the polymer quantity used. The samples were gently stirred in DCM and distilled water. The water layer was collected, and the concentration of carboplatin was determined by UV-Vis spectrometer. Drug loading content and encapsulation efficiency were calculated using the following equation:

$$\begin{array}{cc} Drug\;loading\;content\left(\%\right) & =\frac{Weight\;of\;the\;drug\;in\;NPs}{Weight\;of\;NPs}\times 100\\ & =\frac{C_{extraction}\times V_{extraction}}{W_{extraction}}\times 100 \end{array}$$

$$\begin{array}{cc} Encapsulation\;efficiency\left(\%\right) & =\frac{drug\;loading\;content\times mass\;of\;NPs}{theoretical\;drug\;content}\times 100\\ & =\frac{C_{extraction}\times V_{extraction}÷W_{extraction}}{C_{total}\times V_{total}÷W_{total}}\times 100 \end{array}$$

$C_{extraction}$: concentration of the extracted drug solution

$V_{extraction}$: volume of aqueous buffer used for the extraction

$W_{extraction}$: weight of the NPs used for the extraction

$C_{total}$: concentration of the drug solution that was used in the encapsulation process

$V_{total}$: volume of the drug solution that was used in the encapsulation process

$W_{total}$: weight of the total NPs yield

To visualize the LTPU NPs under light microscopy, larger LTPU NPs were also prepared. The same preparation protocol was followed, except that the initial emulsion was formed using 2000 rpm, followed by 1600 rpm for the secondary emulsion.

### 2.5. In Vitro Cumulative Release Study of CLNPs in PBS

Carboplatin cumulative release from CLNPs in PBS solution was examined by UV-Vis spectrometer. 5 mg of CLNPs were added to each test tube, followed by the addition of 1.1 mL of PBS to each tube. The tubes were then placed in an incubator at 37 °C. After the indicated time intervals, such as 1, 2, 4, 7, and 14 days, each sample was centrifuged at 4000 rpm for 10 min. 1 mL of supernatant from each solution was collected and added to 1 mL of fresh PBS and then placed back in the incubator. The collected supernatant was analyzed by a UV-Vis spectrometer to examine the concentration of released carboplatin. The wavelength maximum was selected at 230 nm to quantify the concentration of carboplatin with a correlation coefficient of 0.995641.

### 2.6. Biodegradation Study of LTPU NPs in PBS

3 mL of PBS was added to the vial containing 5 mg of LTPU NPs and thoroughly suspended in phosphate buffered saline (PBS) with a pH of 7.0. The PBS was changed every 12 h to keep the pH constant. After the suspension was placed in a shaking water bath at 37 °C and 100 rpm for 7 days, the biodegradation of LTPU NPs was investigated by monitoring changes in particle size and morphology using DLS and FE-SEM.

### 2.7. Characterization of the Synthesized LTPU and Its Precursors

Various characteristic techniques, such as FTIR, NMR, GPC, DLS, FE-SEM, and TEM, were performed to identify the structural and morphological changes of LTPU and its precursors and nanoparticles. The homogenizer used for the fabrication of nanoparticles was the MTOPS SR30 homogenizer, capable of high-speed operation ranging from 5000 to 30,000 RPM, equipped with a T20SF nozzle. The chemical structures were characterized by Fourier transform infrared (FT-IR, Cary 600 Series, Agilent Technologies, Santa Clara, CA, USA), proton nuclear magnetic resonance (^1^H-NMR, Unity-Inova 500, Varian Technology, Palo Alto, CA, USA), and carbon nuclear magnetic resonance (^13^C-NMR, AVANCE NEO 500, Bruker Co., Billerica, MA, USA) spectroscopies. ^1^H-NMR and FT-IR spectroscopies were performed three times, with each 256 and 128 scans. Gel permeation chromatography (GPC) analysis was performed to determine the molecular weights and polydispersities of LTPU polymer and PLA-PEG-PLA triblock copolymer using a Waters 1515 pump and Waters 2414 differential refractometer. Styrogel HR3, HR4, and HR5E columns were used with DMF as the eluent at a flow rate of 1 mL/min and a temperature of 35 °C. Linear polystyrene standards from the Shodex standard kit SM-105 were used to obtain a calibration curve.

### 2.8. Characterization of the Fabricated LTPU Nanoparticles

The size of NPs was characterized by dynamic light scattering (DLS) using a Zetasizer Nano-S90 (Malvern Panalytical Korea Co., Ltd., Seongnam, Republic of Korea) equipped with a 633 nm HE-Ne laser, with NPs dispersed in water. DLS analysis was performed five times to obtain mean values with standard deviations. The NPs were examined using transmission electron microscopy (TEM, H-7600, Hitachi, Tokyo, Japan) and field emission-scanning electron microscopy (FE-SEM, SUPRA40VP, Carl Zeiss, Oberkochen, Germany) to confirm their size and morphology. Drug loading content, encapsulation efficiency, and in vitro cumulative drug release study were investigated by an ultraviolet-visible spectrometer (UV-Vis, OPTIZEN 3220UV, MECASYS, Daejeon, Republic of Korea), with a wavelength maximum of 230 nm to quantify the concentration of carboplatin. A fluorescence microscope (80i, Nikon, Tokyo, Japan) and confocal laser scanning microscope (CLSM, LSM 800, Carl Zeiss, Oberkochen, Germany) were used to confirm the morphology of FITC-loaded LTPU NPs.

## 3. Results and Discussion

### 3.1. Characterization of LTHE and DLTHE

**Figure 2:**
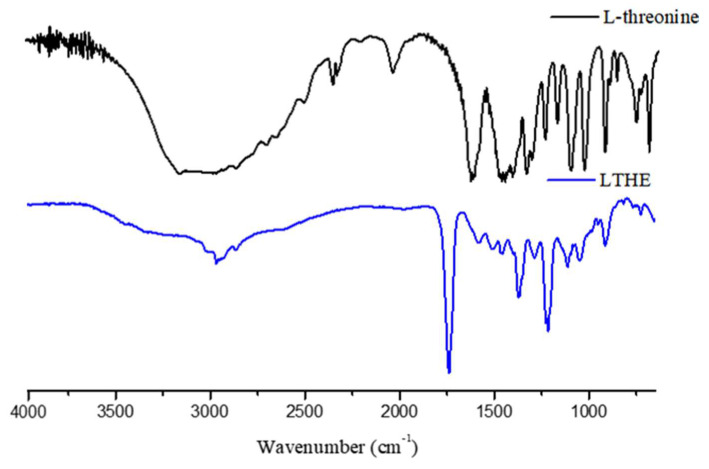
FT-IR spectra of L-threonine and LTHE.

The FT-IR spectrum of LTHE, as shown in Figure 2, exhibits a characteristic C=O stretching peak at 1740 cm^−1^, which was originally observed at 1630 cm^−1^ in L-threonine. This change coincides with the disappearance of the COOH stretching signal, which was previously observed in the range of 3650 to 3000 cm^−1^ due to the esterification reaction between L-threonine and hexanol. The wagging vibration peak of COO^−^ at 700 cm^−1^, the torsional peak of COH at 750 cm^−1^, and the bending peak of COO^−^ at 767 cm^−1^ also disappeared in the spectrum of LTHE as a result of the esterification reaction.

**Figure 3:**
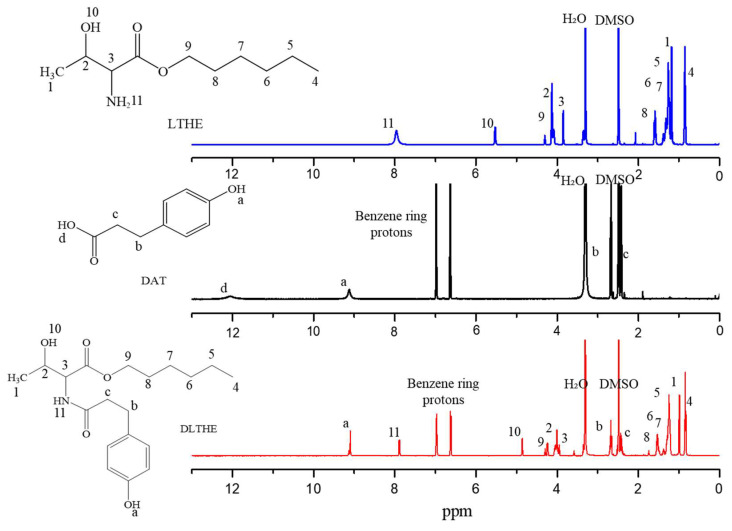
^1^H-NMR spectra of LTHE, DAT, and DLTHE.

The ^1^H-NMR spectra of LTHE, DAT, and DLTHE are shown in Figure 3. In the ^1^H NMR spectrum of LTHE, the methine and methyl peaks appeared between 0.8 and 1.6 ppm, with the methylene peak adjacent to the carbonyl group at 4.2 ppm following esterification.

The ^1^H NMR spectrum of DLTHE, compared to LTHE, exhibits characteristic chemical shifts at 9.10, 6.98, 6.61, 2.67, and 2.43 ppm, corresponding to the hydroxyl proton adjacent to the benzyl group, benzyl protons, and methylene protons adjacent to the benzyl group, respectively. DLTHE also confirmed additional chemical shifts at 4.86, 7.88, 4.12, 1.58, 1.37 to 1.19, 4.07, 3.81, 1.82, and 0.85 ppm, corresponding to the hydroxyl proton, amide proton, methylene protons, methine protons, and methyl proton.

### 3.2. Characterization of PLA-PEG-PLA Triblock Copolymer

**Figure 4:**
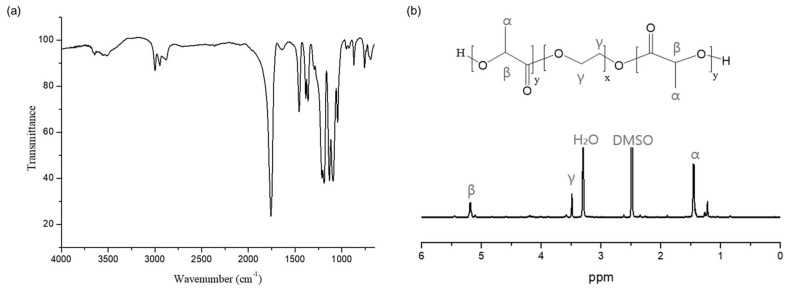
(**a**) FT-IR and (**b**) ^1^H-NMR spectra of PLA-PEG-PLA.

The chemical structure of the PLA-PEG-PLA triblock copolymer was confirmed by FT-IR and ^1^H-NMR analysis. As shown in Figure 4a, the PLA-PEG-PLA triblock copolymer exhibits distinct peaks in its Fourier-transform infrared (FTIR) spectrum. Notably, there are prominent peaks at 3600~3200 cm^−1^, which correspond to the -OH stretching vibrations, providing clear evidence of the presence of polyols with hydroxyl (-OH) groups at both ends. Additionally, a peak at 1760 cm^−1^ is observed, attributed to the C=O stretching vibrations within the PLA blocks. Furthermore, the FTIR spectrum displays the asymmetric and symmetric bending peaks of the methyl groups at 1460 cm^−1^ and 1380 cm^−1^, respectively, in conjunction with C-O stretching peaks spanning the range of 1250 cm^−1^ to 1050 cm^−1^. In the ^1^H-NMR spectrum of PLA-PEG-PLA triblock copolymer shown in Figure 4b, the characteristic chemical shifts for PLA blocks were found at 5.2 ppm for methine (β) protons and 1.4 ppm for methyl (α) protons, respectively. The integral ratio of 1.25:1 between the methine (β) protons of PLA blocks and the methylene (γ) protons of PEG blocks was used to calculate the molecular weight of the triblock copolymer, which was found to be 2800 g/mol. Good agreement was also obtained with the GPC result of 2900 g/mol (Mn) with a polydispersity of 1.57.

### 3.3. Characterization of LTPU

**Figure 5:**
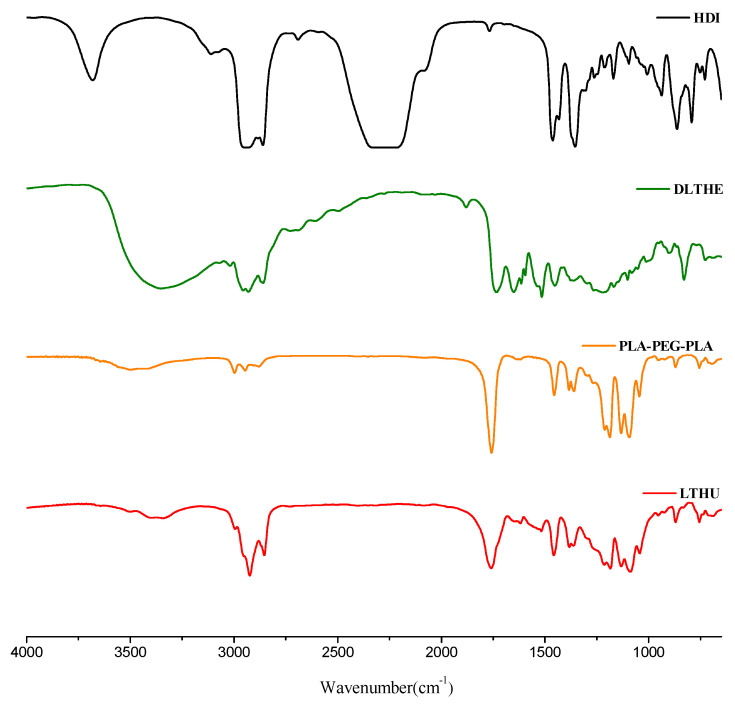
FT-IR spectra of HDI, DLTHE, PLA-PEG-PLA, and LTPU.

The FT-IR spectra of HDI, DLTHE, PLA-PEG-PLA, and LTPU are shown in Figure 5. The FT-IR spectrum of LTPU displayed several characteristic absorption peaks arising from urethane linkages, including amide, carbonyl groups, and C-O stretching peaks at 3600 to 3200 cm^−1^, 1760 cm^−1^, and 1250 to 1050 cm^−1^, respectively. The disappearance of the isocyanate group at 2310 cm^−1^ confirmed the successful synthesis of LTPU. The spectrum also showed additional peaks at 1460, 1380, and 1650 to 1480 cm^−1^, attributed to asymmetric and symmetric bending peaks as well as aromatic C-C stretching peaks. These peaks are due to the aromatic ring of DLTHE, which was used as a chain extender.

**Figure 6:**
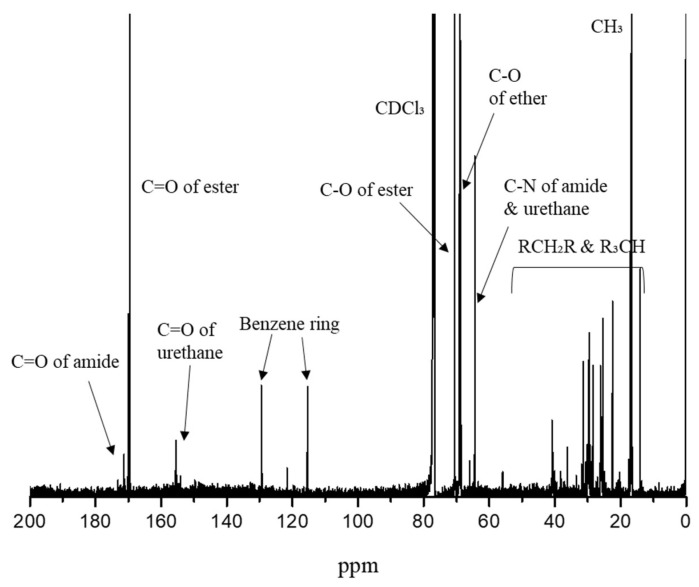
^13^C-NMR spectrum of LTPU.

In Figure 6, the ^13^C-NMR spectrum confirms the following characteristic peaks: amide bond C=O (171 ppm), ester bond C=O (170 ppm), urethane bond C=O (155 ppm), aromatic ring C=C (130 ppm and 115 ppm), ester bond C-O (71 ppm), ether bond C-O (69 ppm), urethane and amide bond C-N (64 ppm), and CH_2_/CH (40–10 ppm) and CH_3_ (17 ppm). Compared to the GPC analysis of the prepolymer, the molecular weight of LTPU increased continuously after chain extension and purification, while the PDI decreased. The molecular weight of LTPU was confirmed to be 28,800 g/mol with a PDI of 1.46.

### 3.4. Characterization of LTPU NPs

**Figure 7:**
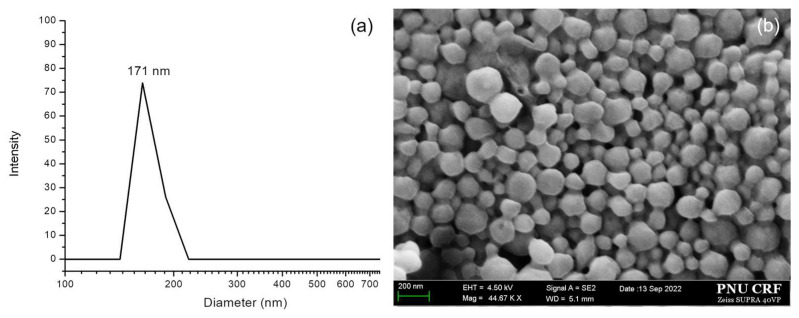
(**a**) DLS and (**b**) FE-SEM image of blank LTPU NPs.

The size and morphology of blank nanoparticles (NPs) have been confirmed by DLS and FE-SEM. As shown in Figure 7a, blank NPs confirmed by DLS have an average diameter of 171 nm, making them suitable for achieving the EPR effect and serving as ideal chemotherapeutic carriers, as nanoparticles with diameters between 100 and 200 nm can passively exit the vascular system while avoiding filtration in the liver and spleen. Good agreement was also obtained with the FE-SEM image provided in Figure 7b, indicating particle sizes within 200 nm with spherical shapes and smooth surfaces.

**Figure 8:**
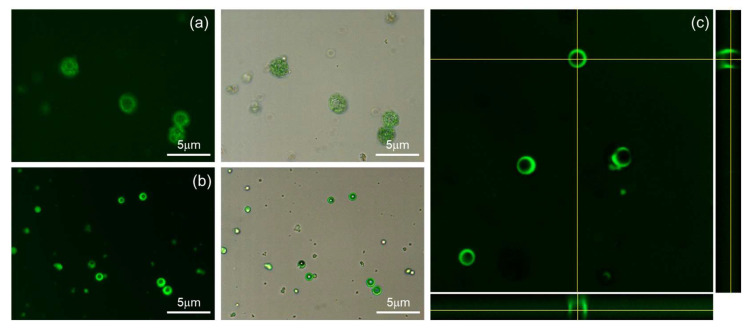
(**a**,**b**) Fluorescence microscope images and (**c**) confocal orthogonal view image of FITC-loaded LTPU NPs.

Confocal microscopy confirmed that FITC-loaded NPs, as displayed in Figure 8a, also exhibited spherical shapes with FITC loaded into the hydrophobic sections of the NPs. Most of the FITC-loaded LTPU NPs, produced using the water-oil-water double emulsion technique, feature several discrete, spherical pockets formed by encapsulating aqueous droplets within the polymer. However, some exceptions exist, as shown in Figure 8b, where certain particles exhibit a ring-like shape in 2D view, indicating the entrapment of FITC. Reconstructed images from confocal slices, as depicted in Figure 8c, confirm the presence of outer ring structures where the tops of NPs are distinguishable from the dark inner circular area. When simulated in a 3D view, these rings give rise to internal shell structures.

**Figure 9:**
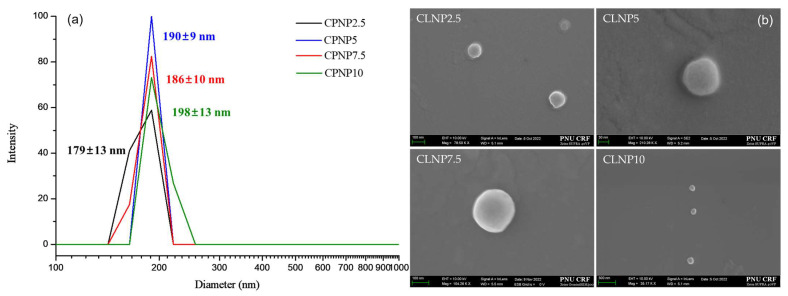
(**a**) DLS and (**b**) FE-SEM images of CLNP2.5, CLNP5, CLNP7.5, and CLNP10.

All variables for the fabrication of carboplatin-loaded LTPU NPs (CLNPs) were held constant, except for the carboplatin concentrations, which ranged from 2.5 to 10 wt%. These variants were named CLNP2.5, CLNP5, CLNP7.5, and CLNP10, respectively. Based on the dynamic light scattering (DLS) results presented in Figure 9a, it is evident that the average particle sizes of CLNPs remained consistent at around 200 nm, irrespective of the varying carboplatin concentrations. Similar findings were also observed in the FE-SEM images shown in Figure 9b, confirming that the average particle sizes were approximately 200 nm with spherical shapes and smooth surfaces.

**Figure 10:**
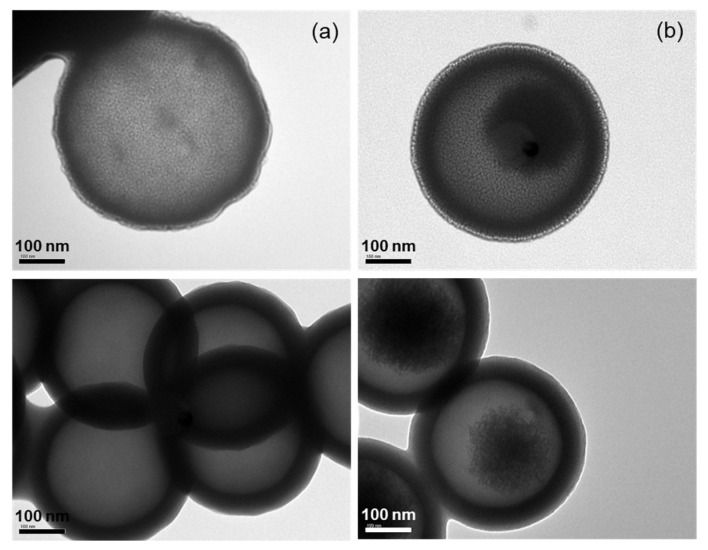
TEM images of (**a**) blank LTPU NPs and (**b**) CLNPs.

The morphology of LTPU NPs, both with and without carboplatin, was also characterized using transmission electron microscopy (TEM). Similar to the results obtained from DLS and FE-SEM, the TEM images of both blank LTPU NPs and CLNPs revealed spherical shapes and smooth surfaces with approximate particle sizes of 400 nm, which are larger compared to the DLS and FE-SEM results. This may be attributed to differences in imaging techniques, sample preparation, and resolution capabilities. In Figure 10b, TEM images of CLNPs reveal small dark inner circular regions, with lighter areas suggesting the distribution of carboplatin within an internal shell of the NP. These observations align with the results from fluorescence microscopies in Figure 8.

### 3.5. Drug Loading and Encapsulation (%), In Vitro Cumulative Release Study and Stability Assay

**Figure 11:**
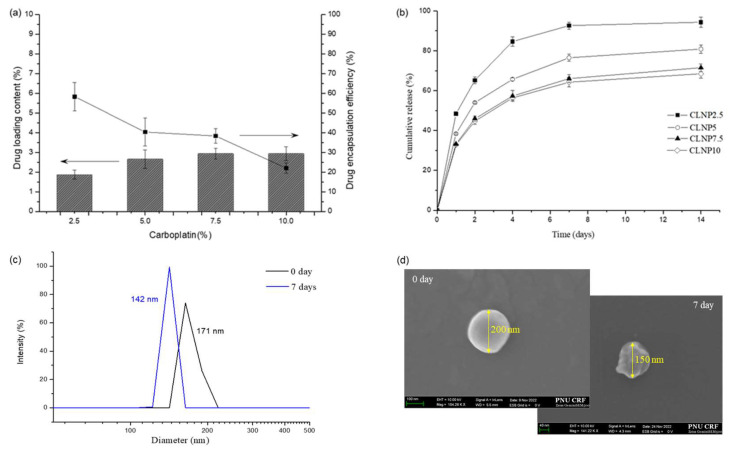
(**a**) Graphs of loading content (bar graph) and encapsulation efficiency (line graph), (**b**) cumulative release results of CLNP2.5, CLNP5, CLNP7.5, and CLNP10, (**c**) changes in diameter, and (**d**) SEM images of LTPU NPs before and after degradation for 7 days in PBS.

As depicted in Figure 11a, the drug loading contents, which provide indirect evidence of where the drug is located within the nanoparticle, for CLNP2.5 and CLNP5 were 1.87% and 2.65%, and the highest drug loading content reached 2.93% at carboplatin concentrations of 7.5 and 10 wt%, respectively. When considering encapsulation efficiency, CLNP2.5 exhibited the highest value at 58.3%, which implies uniform distribution, including within the shell. This value decreased as the amount of drug used increased, declining from 40.3% for CLNP5 to 38.3% for CLNP7.5 and further to 22.0% for CLNP10, respectively.

The cumulative carboplatin release behaviors of CLNPs are illustrated in Figure 11b. All nanoparticles displayed an initial burst release within the first two days, followed by sustained carboplatin release, indicating that, in nanoparticles fabricated via methods such as water-in-oil-in-water (W/O/W) double emulsion, carboplatin can localize in the water phase core or the shell. On day 14, the cumulative release of CLNP2.5 exceeded 90%. In contrast, a different trend emerged with increasing loading concentrations, where the cumulative release of CLNP10 was approximately 60% on the same day. Based on encapsulation efficiency and drug release characteristics, CLNP2.5 appears to be the optimal carboplatin concentration. Figure 11c,d depict changes in both diameter and shape due to degradation over time. The particle size of LTPU NPs decreased by approximately 20%, going from 171 to 142 nm, resulting in rough and irregular surfaces after 7 days. These results indicate that CLNP2.5, with a lower carboplatin load, released most of its encapsulated drugs over 14 days, while CLNP10, with a higher drug load, released only 60% of its encapsulated drugs in the same period. These changes may also reflect the degradation of the shell and drug release from the shell into the surrounding medium, altering the physical dimensions of the nanoparticles. The drug distribution for some of the NPs formed within an internal shell with a short path length to the surface. This result is unexpected and cannot be explained since most drugs using oil-in-water-in-oil emulsion accumulate into hydrophilic pockets of NPs. The NPs with shell loading were expected to have relatively rapid drug release due to the short diffusion path length from the outer surface as compared to the NPs with drugs accumulated in the hydrophobic pockets. Drug distribution throughout the shell of nanoparticles provides a mechanism for the controlled release and protection of the drug. Figure 11 collectively illustrates the effect of the localization of the drug on the release kinetics and the structural integrity of the nanoparticles over time. Finally, the cumulative release and biodegradation behaviors indicate that LTPU NPs undergo surface degradation, with the rate of water penetration into NPs being slower than polymer degradation. This phenomenon is commonly observed in relatively hydrophobic and enzymatically degradable polymers [15,42,43,44].

## 4. Conclusions

A novel biodegradable polyurethane using L-threonine was successfully synthesized and fabricated into NPs. The size of the LTPU NPs has been optimized to be below 200 nm so that they could have EPR properties. While LTPU NPs successfully encapsulated carboplatin and FITC, the internal distribution of the drug and the fluorescence marker has been uniquely distributed into internal shells closed to the surface of the NPs. While some of the NPs have discrete internal pockets of FITC consistent with water-in-oil-in-water emulsion, other NPs seem to have encapsulated FITC and carboplatin within an internal shell. Thus, LTPU has the ability to encapsulate drugs in a distinct way than other polymers. The drug content and the release rate can also be altered by adjusting the drug loading during the fabrication of NPs. Finally, the release rate of carboplatin by LTPU NPs ranges between 60 and 90% and is dependent upon the drug loading. Future studies will focus on investigating in vitro and in vivo cytotoxicities, including the toxicities of both the nanoparticles and their degradation products.

## Figures and Tables

**Table 1:** Formulation of blank and FITC-loaded LTPU NPs by double emulsion method.

	Conc. (mg/mL)	Mass (mg)	Vol (mL)	Mass % *(w/w)*	Vol % *(v/v)*
LTPU	14.78	133	9	95	14.75
PLA-PEG-PLA	7	7	1	5	1.64
5% PVA	-	-	50	-	81.97
	Blank LTPU Nanoparticles				
FITC in H_2_O	0	0	1	-	1.64
Total	-	140	61	100	100.00
	FITC-loaded LTPU Nanoparticles				
FITC in H_2_O	0.5	0.5	1	0.36	1.64
Total	-	140.5	61	100.00	100.00

## Data Availability

The raw data supporting the conclusions of this article will be made available by the authors on request.
